# A short review on the aetiology and pathophysiology of alcoholism

**DOI:** 10.1186/1744-859X-8-10

**Published:** 2009-05-14

**Authors:** George Moussas, Christos Christodoulou, Athanassios Douzenis

**Affiliations:** 1 Second Psychiatry Department, Athens University Medical School, Attikon General Hospital, Athens, Greece

## Abstract

Alcoholism is a chronic remitting and relapsing condition; its aetiology and pathophysiology remains largely obscure despite recent advances. This review summarises the current knowledge about the causation (biological or psychological) of alcohol addiction. This involves heredity, candidate genes, alcohol metabolism regulation and the influence of alcohol in the pathophysiology of the different neurotransmitter systems. Alcohol addiction is a multifactorial phenomenon where personality structure, individual state of mind and social influences are in constant interaction with brain neurobiology and pathophysiology. This disorder influences different sexes in different ways and causes major problems, especially in developed societies.

## Background

It is well established that alcoholism is a chronic condition and a major public health issue. Alcoholism presents as a continuum with different presentations among different individuals. This continuum starts from habitual consumption, to problematic use/abuse, to severe abuse with physical problems and finally addiction. It is certain that the physical and psychological problems that accompany the pathological relationship with alcohol increase with the quantity consumed and the frequency of use. Alcohol addiction is a multifactorial disorder that runs transgenerationally [1].

## Genetic components of alcoholism and subtypes of alcoholism

Genetics have an important and critical contribution in the development of alcohol abuse. Despite significant indications for the involvement of the genetic factor the risk that is inherited remains unknown [2-4].

### Twin studies

Twin studies underline the significance of the genetic influences. Overall, the comparison of monozygotic (MZ) and dizygotic (DZ) twins has shown a greater concordance for addiction in the monozygotic group. Twin studies in Sweden reported a 54% alcoholism prevalence between MZ and 28% in DZ twins. Epidemiological studies in groups of twins have shown that the risk of hereditary transmission for alcoholism ranges between 0.52 and 0.64 with no significant difference between the sexes [5]. However, there are different genetic influence for the inheritance of alcohol abuse and different for alcohol dependence. The liability for alcohol abuse in male subjects is attributed to genetic and shared environmental factors. For female alcohol abuse, liability is attributed to shared and non-shared environmental factors with no evidence of genetic influence [6] though women might carry a lower genetic risk for alcohol dependence [7]. Overall, the results of the majority of twin genetic studies support the existence of significant genetic factors that predispose individuals to the development of alcohol related problems [8,9].

### Family studies

There have been several family studies on alcoholism that have provided important knowledge about the inheritance and the predisposition to alcoholism through the generations. From as early as 1940 Jellinek and Jolliffe suggested the existence of a familial and a non-familial form of alcoholism [10].

There are two possible ways of transmission: either there is genetic heterogeneity with two distinct subtypes (type I with low and type II with high genetic influence), or a mixed pattern of transmission including a dominant gene with a multifactorial substrate [6].

### Adoption studies

Children of alcoholics that had been adopted away and had no contact with their biological parents offer the opportunity to answer the 'nature vs nurture' question. Adopted children with an alcoholic biological parent (usually the father) develop alcohol addiction more frequently compared to a control group of adopted children with no alcoholic parent [7,8]. More recent studies have also shown similar results. In these studies the percentage of alcoholism in boys with alcoholic parents (18%) is four times greater than the percentage of a similar group of boys with non-alcoholic parents regardless of whether the children were brought up by the their biological parents or were adopted, and the risk ratio vary between 1.6 and 3.6 in males, and between 0.5 and 6.3 in females [11,12].

In 1992 Babor *et al. *confirmed the existence of two alcohol addiction subtypes, which are probably related to the causation of alcoholism: group A and group B, which are similar to type I and type II alcoholism [13].

## Biological markers of alcohol consumption

### Candidate gene studies: alcohol-metabolising enzymes

Amongst the variables that enhance the risk of developing alcoholism, the genes responsible for the liver enzymes are believed to be related to an increased risk for alcohol dependence. This is the case even when these genes are not directly associated with the neuropharmacological effects of alcohol.

In alcohol metabolism, ethanol is metabolised to acetic acid, from three different enzymatic systems in the liver cells:

1. Alcohol dehydrogenase (ADH) metabolising alcohol to acetaldehyde.

2. The microsomal oxidation system (MEOS) of ethanol that turns ethanol into acetaldehyde.

3. Aldehyde dehydrogenase (ALDH) that transforms acetaldehyde into acetic acid [14].

Genotyping these enzymes can explain individual differences in the concentration and metabolism of ethanol after alcohol consumption. It has been hypothesised that these differences play a crucial role in the development (or not) of alcohol addiction [14,15].

Genetic studies conducted in various ethnic groups have confirmed that certain allele variations of ADH offer strong protection against alcohol addiction. Alcoholics are less likely to possess allele variations of ADH and ALDH, which metabolise alcohol, when compared to non-alcoholics. Findings from various studies suggest that the strong genetic influence found in alcoholism is related to inherited gene variations of enzymes that alter alcohol metabolism. These gene variations contribute to the development of alcohol addiction through a mechanism that is not as yet known [15-20].

### Candidate gene studies: neurotransmitter genes

In addition to the enzyme genes, neurotransmitter genes have been associated with increased risk for alcohol dependence. Psychotropic substances (including alcohol) modify the neurophysiological chemical changes that take place in the brain. The indications for a genetically defined predisposition responsible for alcohol dependence are examined in the field of different candidates for neurotransmitter genes [21,22]. These genes influence the vulnerability of developing dependency syndromes, such as alcoholism. One of the genes studied is the D2 dopamine receptor protein (genetic locus DRD2). Dopamine is generally known to play a significant role in the substance dependence development, and one of its receptors is the DRD2 receptor. Alcohol acts in the mesolimbic striatum, altering the synaptic function thus causing increased dopaminergic activity [22,23].

Alcohol acts on many neuroreceptor systems and, as already mentioned, alcohol addiction differs from other addictions in that it has no known receptor system in the brain. It also alters the activity of serotonin receptors (5 HT3). Additionally it acts on the nicotinic receptors. It modifies the γ-aminobutyric acid (GABA)ergic neurotransmitter (type A: GABA-A), which is an inhibitory neurotransmitter, and also acts on the *N*-methyl-D-aspartic acid (NMDA) receptors of glutaminergic neurotransmission and on the NMDA subgroups on the glutaminergic receptor-stimulating neurotransmitter. Furthermore alcohol inhibits the δ-opiate receptors while long-term exposure to the alcohol increases the density of μ and δ receptors and generally increases endogenous opiate levels in opioid neurotransmission [23,24].

Additionally, serotonin also seems to play a significant role in the regulation of alcohol consumption. The serotonin transporter (5-HTT) intercedes to the presynaptic serotonin reuptake and thus serotoninergic neurotransmission is concluded. The gene that codes the 5-HTT protein shows a polymorphism having two common allelic genes that are different in length, L (long) and S (short). Various conditions related to addiction such as tolerance, sensitivity, dependence and so on, are the results of a molecular and cellular adaptation which takes place in specific brain areas as a results of repeated exposure to alcohol [24,25]. The exact neurobiological changes at the pharmacological level all the above-named actions provoke, and how they are related to the compulsive substance-seeking phenomenon (craving) are unknown.

Alcohol abuse and substance abuse in general is related to the immediate reward the substance offers, as well as the reinforcement caused by the repeated substance use. The mesolimbic pathway is considered crucial for the reward procedure. This pathway starts from the ventricular area, which is an area rich in dopamine in the midbrain (the ventral tegmental area (VTA)) [1,22]. The stimulating addictive substances act directly on the metasynaptic dopamine receptors, whilst non-stimulating ones, such as alcohol, act via the mesolimbic pathway through various receptor systems. Alcohol acts on the mesolimbic pathway modifying neuronal stimulation by interacting with ion channels and ionic receptors, affecting polarisation [26]. Alcohol increases the sensitivity of 5-HT3 serotonin receptors and this seems to be implicated in alcohol abuse/dependency development. Chronic alcohol use does not influence just one neurotransmitter but almost all neurotransmitter systems [27,28]. These actions can be categorised as following:

• Reward craving, which is related to dopaminergic dysfunction as well to dysfunction of opioid receptor transmission.

• Relief craving, which is related to GABAergic and glutaminergic transmitter dysfunction.

• Obsessive craving, which is related to loss of control and is based to serotoninergic dysfunction [29].

A similar effect appears through modification of the functional activity of the proteins that are already inside the neuron or through the influence of alcohol on their 'real' quantity in the neuron [30].

## Psychological causes

Personality has also attracted interest in relation to its contribution to the development of alcoholism, despite the fact that the theory of a distinct type of personality that leads to alcohol dependency has been rejected. Idiosyncrasy and personality traits have been intensively studied in order to investigate their association with vulnerability for alcohol dependence [30,31]. Previous studies concluded that some traits such as low self-esteem had a positive relation to alcoholism. Recent studies comparing boys with an alcoholic parent and boys with a non-alcoholic parent found no differences with regards to personality traits such as innovation seeking, damage avoidance and reward dependence [32]. Behavioural double inhibition is regarded as weakness or lack of desire for a person to inhibit these drives. Antisocial behaviour is considered not only to be related more than any other behavioural disorder to alcohol dependence, but it also may predict it. In this complicated relationship, genetic and environmental factors also make a contribution. According to theories alcohol abuse can be considered as a habit acquired through three basic procedures:

1. Modelling.

2. Operant conditioning or type II dependence according to Pavlovian theory.

3. Basic conditioning or type I, according the same theory.

As far as modelling is concerned, the model of the parents, especially the parent of the same sex seems to be significant. Operant conditioning is related to the increase of satisfaction and the decrease of unpleasant feelings (uneasiness, irritability, anxiety), through alcohol consumption [33].

Finally, basic conditioning in relation to the generalisation mechanism, the atmosphere of the bar or the public place where the individuals drink, can potentially lead to the same effect as alcohol consumption; that is, relief from anxiety. Generalisation may lead to a variety of new stimuli that reinforce the habit of alcohol consumption [32].

These theories not only offer information about diagnosis, but also the theoretical basis for developing therapeutic techniques.

## Conclusion

Although we now know much more about the influence of alcohol on the central nervous system, the mechanisms of action remain under discussion. One of the main problems is related to the fact that alcohol influences more than one neurochemical system of the brain. These influences are different if the quantity of alcohol consumed is large or small. In some cases, this leads to contrasting effects (toxicity versus withdrawal syndrome). Chronic alcohol exposure may lead to changes in many significant brain functions. In addition, psychological factors and mechanisms are in constant interaction with the biological background, the genetic influence and the sociocultural environment, creating new clinical results and various study areas.

## Competing interests

The authors declare that they have no competing interests.

## Authors' contributions

GM and AD designed the study and wrote the paper. CC collected the references and analysed them.
